# Investigation into the communication between unheated and heat-stressed *Caenorhabditis elegans* via volatile stress signals

**DOI:** 10.1038/s41598-022-26554-8

**Published:** 2023-02-24

**Authors:** Liangwen Chen, Yun Wang, Xiuhong Zhou, Ting Wang, Huimin Zhan, Fei Wu, Haolan Li, Po Bian, Zhongwen Xie

**Affiliations:** 1 State Key Laboratory of Tea Plant Biology and Utilization, School of Tea and Food Sciences and Technology, Anhui Agricultural University, Hefei, 230036 People’s Republic of China; 2 Key Laboratory of Bioresource and Environmental Biotechnology of Anhui Higher Education Institutes, School of Bioengineering, Huainan Normal University, Huainan, 232001 People’s Republic of China; 3 Key Laboratory of High Magnetic Field and Ion Beam Physical Biology, Hefei Institutes of Physical Science, Chinese Academy of Sciences, Hefei, 230031 People’s Republic of China

**Keywords:** Ecology, Environmental social sciences

## Abstract

Our research group has recently found that radiation-induced airborne stress signals can be used for communication among *Caenorhabditis elegans* (*C. elegans*). This paper addresses the question of whether heat stress can also induce the emission of airborne stress signals to alert neighboring *C. elegans* and elicit their subsequent stress response. Here, we report that heat-stressed *C. elegans* produces volatile stress signals that trigger an increase in radiation resistance in neighboring unheated *C. elegans*. When several loss-of-function mutations affecting thermosensory neuron (AFD), heat shock factor-1, HSP-4, and small heat-shock proteins were used to test heat-stressed *C. elegans,* we found that the production of volatile stress signals was blocked, demonstrating that the heat shock response and ER pathway are involved in controlling the production of volatile stress signals. Our data further indicated that mutations affecting the DNA damage response (DDR) also inhibited the increase in radiation resistance in neighboring unheated *C. elegans* that might have received volatile stress signals, indicating that the DDR might contribute to radioadaptive responses induction by volatile stress signals. In addition, the regulatory pattern of signal production and action was preliminarily clarified. Together, the results of this study demonstrated that heat-stressed nematodes communicate with unheated nematodes via volatile stress signals.

## Introduction

Current knowledge shows that communication within and between organisms, i.e., bacteria, protozoa, animals, fungi and plants, is essential^1^. Compared to biocommunication in bacteria, fungi, plants and viruses, animals can not only determine self and nonself organisms by relying on volatile substances such as pheromones but also transport various signals via tactile behavior, vocal sounds and visual gestures^1^. Since allelopathic effects are easily confused by behavioral and instinctive responses, it is difficult to prove whether chemical stresses or warning signals can be transmitted in the animal world^2^. It has been reported that when faced with high density or food shortage stress, *Daphnia* species can suppress their reproduction to protect the survival of the entire population by sensing chemical stress signals at the population level^3^. In mammals, Surinov et al*.* showed that irradiated mice generate stress signals and transmit these signals to unirradiated mice^4^. Recent studies have further demonstrated the communication of radiation-induced stress signals in rainbow trout (*Oncorhynchus mykiss, W*), zebrafish (*Danio rerio*)*, z*ebrafish embryos, and *Caenorhabditis elegans* (*C. elegans*)^2,5–7^. It is important to explore the stress signals communicated in vivo between organisms to ensure their survival.

The nematode *C. elegans* has emerged as an important animal model and has been widely used to investigate the innate immune system, signal transduction, development, and nervous system, mainly due to its easy maintenance, short lifespan of approximately 15–21 days, small body size, and abundant mutant strains. Importantly, the results of trials on *C. elegans* can be predictive of outcomes in higher organisms^8–12^. Of the 959 somatic cells of the hermaphrodite nematode, 302 are neurons, including 12 pairs of amphid chemosensory neurons that mediate the detection of various water-soluble and volatile chemicals in the environment^8,13,14^. Because of the absence of auditory and visual senses, interorganismal communication relies on chemosensory neuron input in *C. elegans.* It has been reported that a high-density population of *C. elegans* sensed ascarosides through several types of chemosensory head neurons to induce the development of the dauer larval stage, as well as to control various behaviors, including sexual attraction^15–21^, avoidance, aggregation^20,22,23^, olfactory plasticity^24,25^, lifespan^26^, and stress resistance^26,27^. Recent research by Yu et al*.* has shown that irradiated *C. elegans* can communicate stress signals to unirradiated *C. elegans* by the cysteine protease CPR-4, which is secreted from animals irradiated with UVC or gamma rays^7^. In addition to these water-soluble cues, *C. elegans* also relies on their olfactory sensory neurons to sense a large number of volatile compounds, such as natural products of bacterial metabolism^27–30^.

Recently, our group demonstrated that *C. elegans* irradiated by 25 Gy gamma-irradiation released volatile stress signals to induce radioadaptive responses (RAR) in unirradiated naive *C. elegans*^31^. However, to date, there are few reports about the volatile signals in *C. elegans* under natural stress*.* To determine whether *C. elegans* produces and responds to these volatile stress signals, we used a previously developed cocultivation system^31^ in which RAR of embryonic lethality were used as a physiological/developmental endpoint to evaluate the presence of volatile stress signals.

## Results

### Heat stress-induced production of volatile stress signals in *C. elegans*

**Figure 1:**
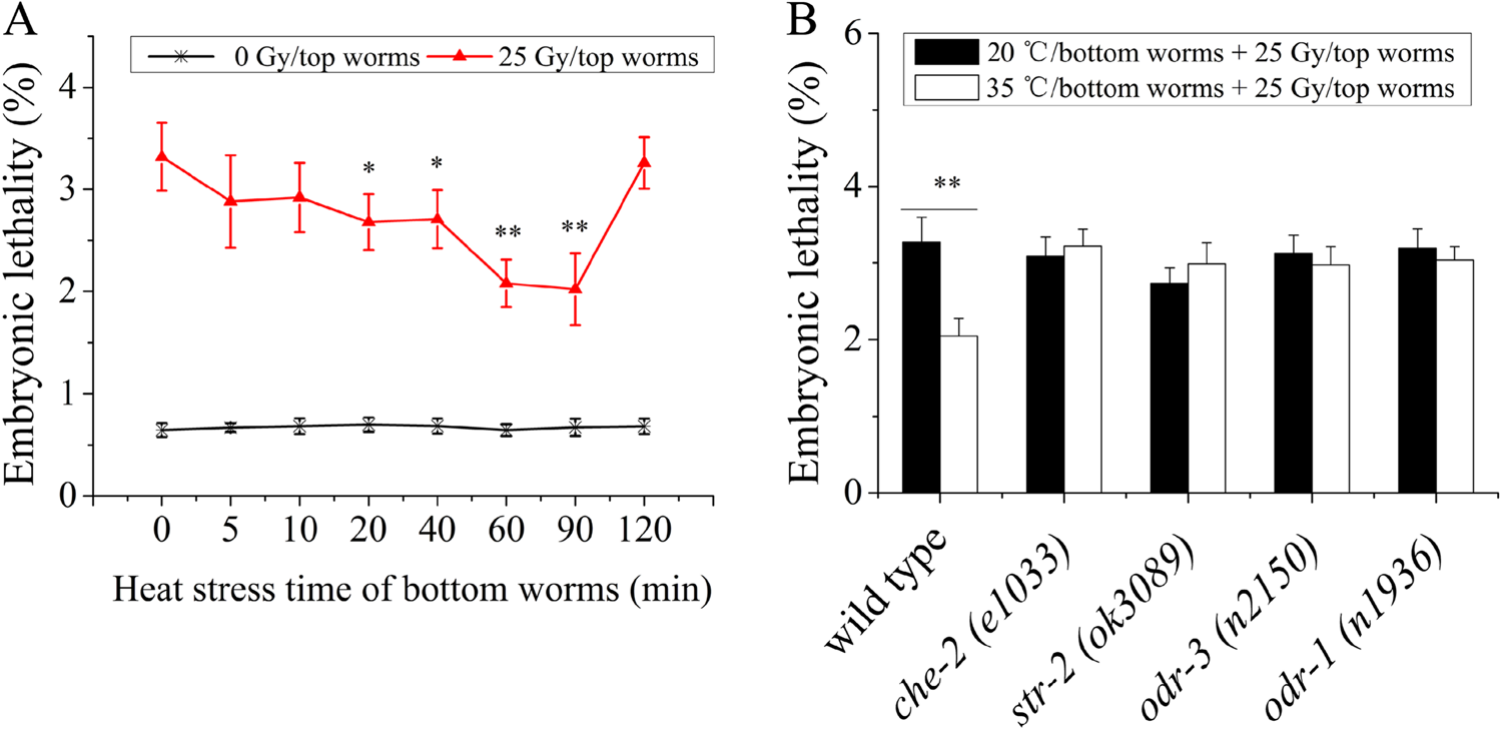
Heat-stressed bottom worms can induce RAR of embryonic lethality in top worms via the volatile stress signals. (**A**) The change of embryo lethality of top worms after coculture with bottom worms subjected to 35 °C heat exposure; (**B**) Embryonic lethality of top worms whose sensory nerve function is absent (*che-2, str-2, odr-3, and odr-1*) after coculture with the bottom worms (N2). Results are means ± SD (n = 5, *P < 0.05, **P < 0.01).

To confirm the existence of volatile stress signals released by heat-stressed *C. elegans*, we used a coculture experimental system (Fig. 7) in which two Petri dishes (“top” and “bottom”) were sealed together with parafilm. The embryonic lethality of the top worms was used as a cue to detect volatile stress signals. First, the bottom worms were heated at 35 °C for 0–2 h and were then cocultured with the top worms for 6 h. The embryonic lethality in top naive worms (0 Gy) was examined. The results indicated that the embryonic lethality rate was not affected in top naive worms (in all cases, *P* > 0.05), as shown in Fig. 1A. Our group has recently demonstrated that *C. elegans* irradiated with 25 Gy of gamma-irradiation released volatile stress signals to induce RAR in naive unirradiated *C. elegans*^31^. We then hypothesized that the heat stress-induced volatile signals might induce RAR in top worms. To verify this possibility, we chose 25 Gy as the challenge dose after cocultivation of the top worms for 6 h. As shown in Fig. 1A, interestingly, heat exposure of the bottom worms for 20 min, 40 min, 60 min, or 90 min significantly reduced the embryonic lethality rate in the top worms irradiated with 25 Gy (in all cases, *P* < 0.05). This result indicated that heat-induced volatile signals may be involved in this protective effect. Moreover, we also found that the heat-induced volatile signals did not significantly affect the per plate brood size of the top worms, as shown in Table 1. Therefore, we focused on the use of a combination of 1 h/35 °C (bottom) + 25 Gy (top), unless otherwise specified. *C. elegans* can grow at temperatures ranging from 12 to 25 °C and is subjected to heat stress once above 25 °C^32^ To verify that different heat-stress conditions can induce the production of volatile signals, the bottom worms were heated at 20/25/30/35 °C for 1 h. The results showed that the heat exposure of the bottom worms at 30 and 35 °C significantly reduced the embryonic lethality rate in the top worms irradiated with 25 Gy (in all cases, *P* < 0.05), while the growth of the bottom worms at normal temperature did not affect the embryonic lethality rate of the top worms (in all cases, *P* > 0.05), as shown in Fig. S1. It has been reported that *C. elegans* is able to find food by sensing the smell of bacterial metabolites^33^. To eliminate the possibility that the volatile signals come from *Escherichia* *coli* under heat stress, *E. coli* (OP50) on the bottom dishes was heated at 35 °C alone and had no effect on the embryonic lethality rate of the top worms (Fig. S2). *Caenorhabditis* *elegans* can sense various volatile chemicals via the olfactory nerve8. Our data showed that embryonic lethality was not changed when sensory nerve function was absent (*che-2*, *str-2*, *odr-3*, and *odr-1*) in the top worms (in all cases, *P* > 0.05), as shown in Fig. 1B. The above results demonstrated that heat-stressed worms could produce volatile stress signals that initiated RAR in neighboring nematodes.

**Table 1:** Embryonic lethality of top worms after laying eggs for 36 h.

Heat stress time (min)	Number unhatched eggs (mean ± sd)	Total eggs (mean ± sd)	Embryonic lethality (%) (mean ± sd)
0	4.70 ± 0.30	142.05 ± 6.45	3.33 ± 0.33
5	4.17 ± 0.63	144.95 ± 5.68	2.88 ± 0.45
10	4.40 ± 0.43	151.00 ± 6.51	2.91 ± 0.34
20	3.76 ± 0.46	140.17 ± 4.50	2.68 ± 0.27
40	3.84 ± 0.57	141.23 ± 10.21	2.71 ± 0.28
60	3.12 ± 0.43	146.94 ± 6.67	2.12 ± 0.37
90	3.04 ± 0.47	150.20 ± 7.20	2.02 ± 0.35
120	4.78 ± 0.43	146.53 ± 7.34	3.26 ± 0.25

### Effects of nematode developmental stages and culture density on the production of heat-stress volatile signals

**Figure 2:**
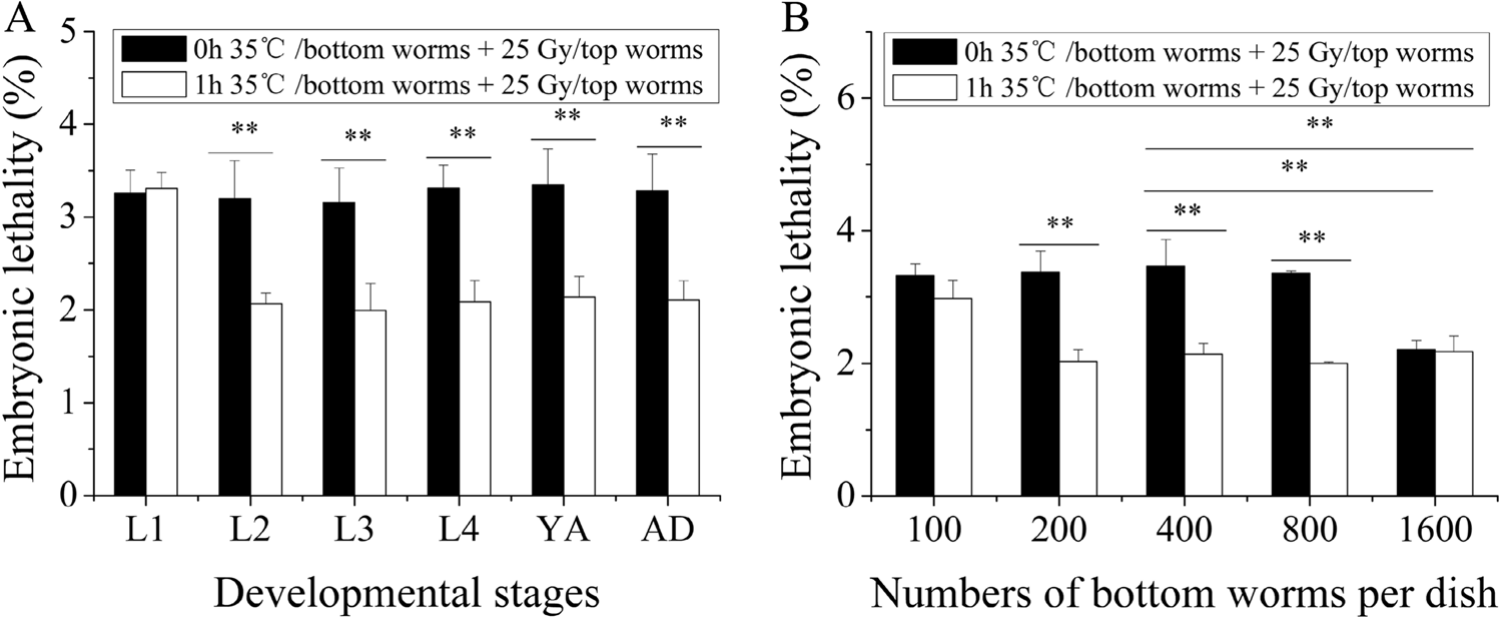
The production of the volatile stress signals in heat-stressed worms depends on their developmental stage and culture density. (**A**) Effect of developmental stage on the production of the volatile stress signals; (**B**) Effect of the culture density on the production of the volatile stress signals. Results are means ± SD (n = 5, **P < 0.01).

*The life cycle of* *C. elegans* *comprises the embryonic stage, four* larval stages (L1–L2–L3–L4), a young adult stage and adulthood. Some chemical signals secreted by *C. elegans* are closely related to its developmental stage and density^34^. Therefore, we first detected the effect of the developmental stages of the bottom worms on the production of volatile stress signals. As shown in Fig. 2A, only heat-stressed L1 worms did not alleviate embryonic lethality in the top worms (in all cases, *P* > 0.05). Heat stress during other developmental stages reduced the embryonic lethality rate in the top worms (in all cases, *P* < 0.01). Furthermore, we examined the effect of the culture density of the bottom worms on the production of volatile stress signals. As shown in Fig. 2B, the RAR of embryonic lethality was observed in the top worms when the culture density of the bottom worms was 200, 400, and 800 worms per dish (wpd) (in all cases, *P* < 0.01). However, the RAR of embryonic lethality was prevented when the culture density of the bottom worms was 100 wpd (*P* > 0.05). Therefore, the L3 developmental stage and a culture density of 400 wpd for the bottom worms were selected in the following experiments unless otherwise specified.

### Time course of the heat-stress volatile stress signals 

**Figure 3:**
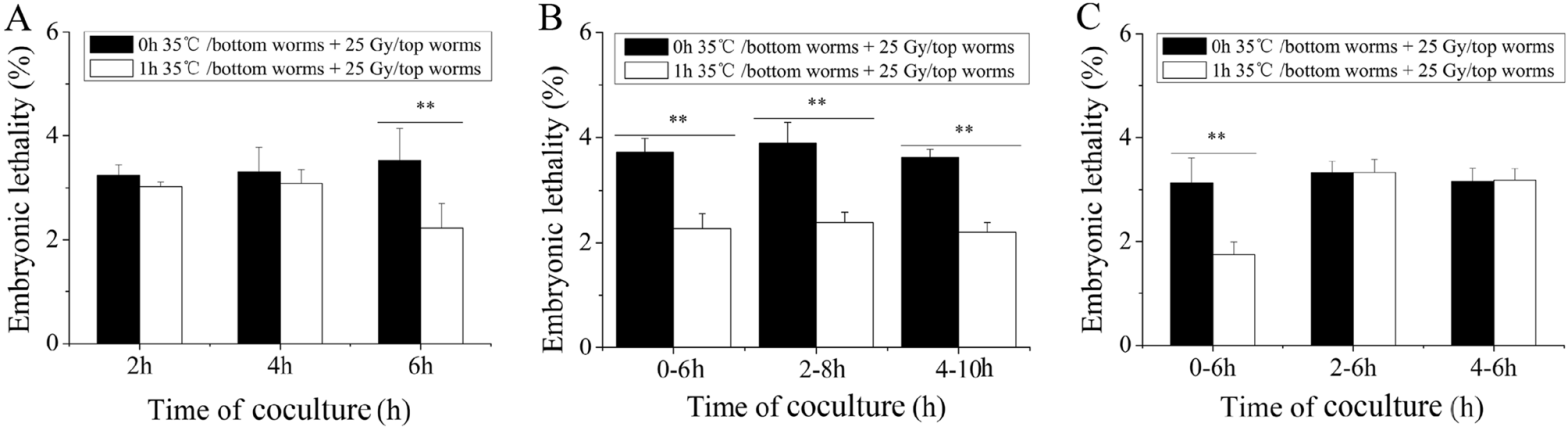
The induction of RAR in the top worms depends on the coculture time of the bottom heat-stressed worms. (**A**) Embryonic lethality of the top worms removed from the coculture system at the designated time point after coculture; (**B**) Embryonic lethality of the top worms moved into the coculture system for 6 h at the designated time point after the heat shock of bottom worms; (**C**) Embryonic lethality of the top worms moved into the coculture system at the designated time point after the heat shock of bottom worms. Results are means ± SD (n = 5, **P < 0.01).

To determine the time course of the volatile stress signals induced by heat stress in the bottom worms, these worms were removed from the coculture system after 2 h, 4 h, and 6 h and then transferred to the coculture system for 6 h at 0 h, 2 h, and 4 h after heat stress. The embryonic lethality rate of the top worms was alleviated only after 6 h of coculture with the heat-stressed bottom worms (in all cases, *P* < 0.01), as shown in Fig. 3A, indicating that it may take approximately 6 h for bottom worms to produce enough volatile stress signals for RAR induction after heat stress. To exclude the influence of heat stress, the bottom worms were transferred to the coculture system at 0 h, 2 h, and 4 h after 6 h of heat stress, and then the top worms were exposed to 25 Gy. As shown in Fig. 3B, the RAR of embryonic lethality was induced in the top worms (in all cases, *P* < 0.01), suggesting that the induction of RAR in the top worms requires continuous exposure to volatile signals for 6 h. Next, the bottom worms were transferred to the coculture system at 0 h, 2 h, and 4 h after heat stress, and then the top worms were exposed to 25 Gy at the 6th hour. The results showed that the RAR of embryonic lethality in the top worms only occurred when transfer of the bottom worms at 0 h after heat stress occurred (*P* < 0.01) (Fig. 3C), suggesting that volatile stress signals were produced immediately after heat stress in the bottom worms.

### Effect of heat-stress volatile signals on germ cells of the top worms

**Figure 4:**
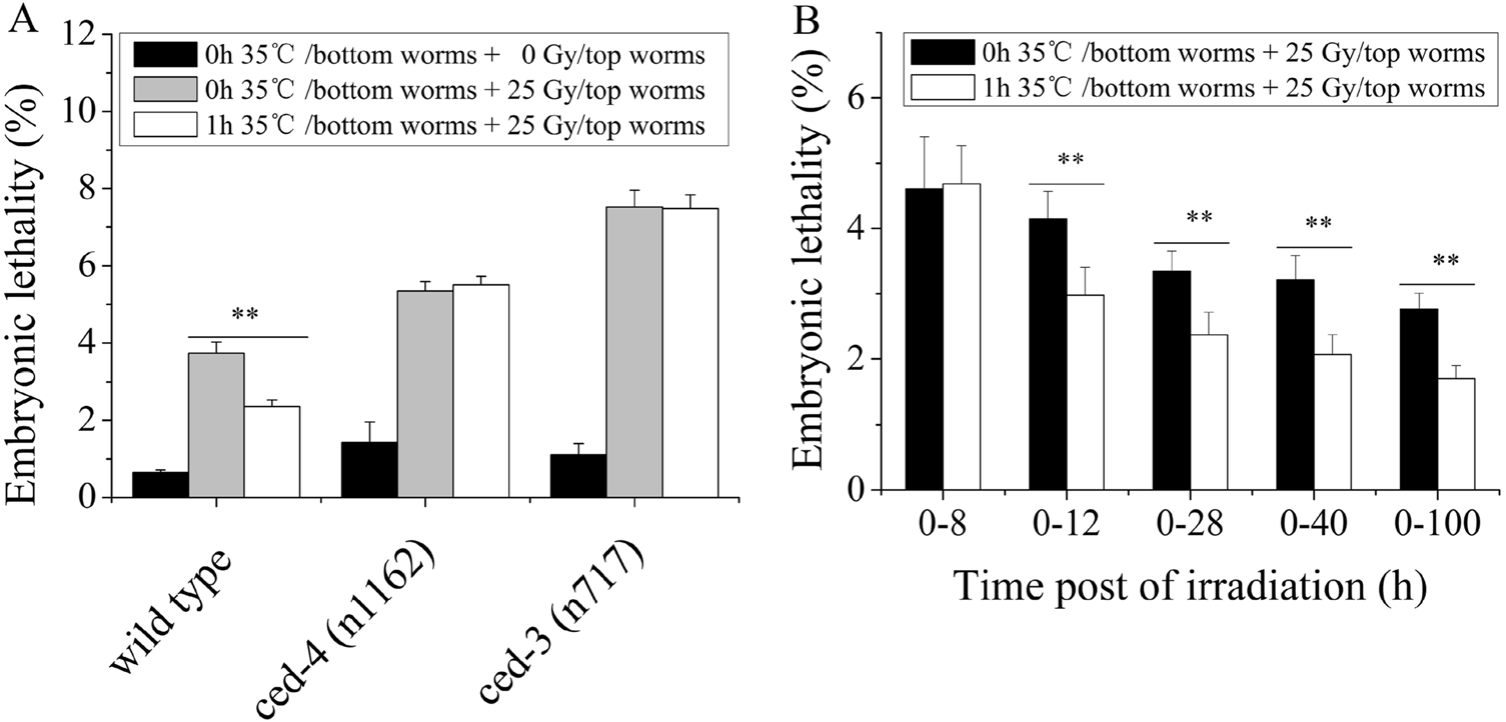
The heat-stress volatile signals mainly acts on the mitotic proliferating cell and meiosis cell of the gonads of top worms. (**A**) Embryonic lethality of the top worms whose apoptosis function is absent (*ced-3* and *ced-4*) after coculture with the heat-stressed worms (N2); (**B**) Embryonic lethality of the top worms at different time points following challenge irradiation after coculture with the heat-stressed worms. Results are means ± SD (n = 5, **P < 0.01).

Hermaphroditic nematodes have both spermatheca and a uterus and generate progeny primarily through self-fertilization^35^. Our results showed that heat stress-induced volatile signals could cause the top worms to initiate RAR to alleviate embryonic lethality, which raised the question of whether RAR induced by volatile stress signals occurs in sperm cells or in female germ cells. To determine this, cell death-defective *ced-3* and *ced-4* worms^36^ were used as the top worms, considering that germ cell apoptosis occurs only during oocyte production in adult hermaphrodites^37^. RAR induction in the top worms was apparently prevented by the absence of *ced-4* and *ced-3* (in both cases, *P* > 0.05), as shown in Fig. 4A, suggesting that volatile stress signals might primarily affect the female germ cells of the top worms. Moreover, in nematodes, embryogenesis proceeds through several stages, the fertilization stage, the fully grown oocyte stage, the late pachytene stage, the pachytene nuclei stage, and the mitotic stage, each with a fixed developmental time^38^. To examine which stage of the female germ cells is specifically affected by volatile stress signals, the embryonic lethality of the top worms was detected at different time points after irradiation. As shown in Fig. 4B, the embryonic lethality of the top worms was reduced for 0–12 h, 0–28 h, 0–40 h, and 0–100 h after irradiation, but the embryonic lethality of the top worms was not reduced for 0–8 h after irradiation. Existing oocytes were completely exhausted within ~ 8 h after egg production^38^ (here, ~ 8 h after irradiation), indicating that volatile stress signals might mainly act on the meiotic and mitotic proliferating zones of the gonads.

### Role of DNA damage response (DDR) in the induction of RAR by the heat-stress volatile signals

**Figure 5:**
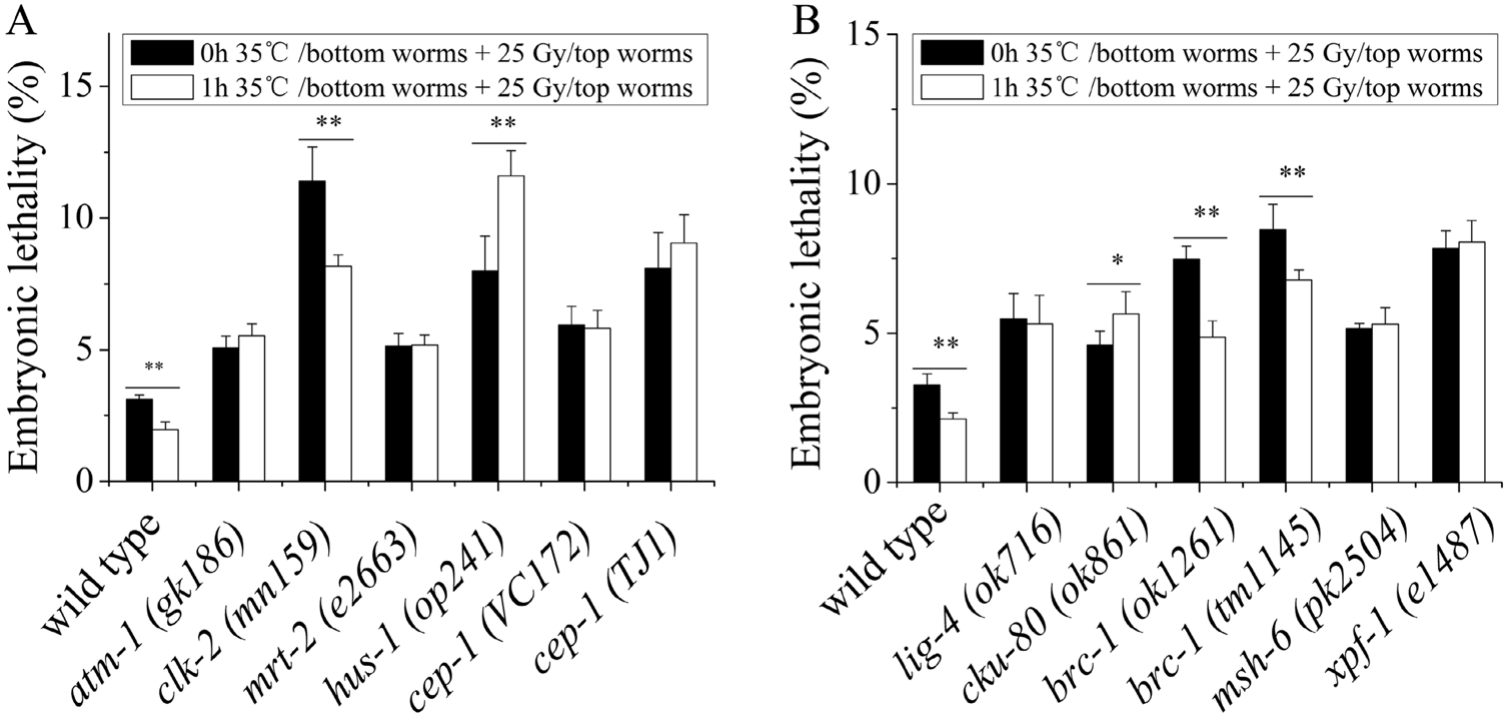
DDR is involved in the regulation of RAR induced by heat-stress volatile signals. (**A**) Embryonic lethality of the top worms whose DNA damage checkpoint function (*atm-1, clk-2, mrt-2, hus-1, and cep-1*) is absent after coculture with the heat-stressed worms (N2); (**B**) Embryonic lethality of the top worms whose DNA repair function (HR, NHEJ , MMR, and NER) is absent after coculture with the heat-stressed worms (N2). Results are means ± SD (n = 5, *P < 0.05, **P < 0.01).

The integrity of the genome is critical to the health of individuals and the continuation of species. To maintain the fidelity of the genome, damaged genomes need to be monitored and repaired in organisms^39^. In response to ionizing radiation, *C. elegans* initiates the DNA damage response (DDR), which includes the checkpoint pathway, DNA repair, cell cycle arrest, and apoptosis, to remove genetically damaged cells that might harm the organism^40^. Therefore, we asked whether DDR plays an important role in RAR induction via volatile stress signals. To investigate this issue, several mutant worms that lost functions in the checkpoint pathway and DNA repair were used as the top worms. For the DNA damage checkpoints, mutant worms (*atm-1, mrt-2, clk-2, cep-1* and *hus-1*) were used for testing. The results showed the embryonic lethality of the mutant worms, including *atm-1, mrt-2*, and *cep-1* (*P* > 0.05). However, the embryonic lethality of the *hus-1* and *clk-2* mutants was reduced significantly (*P* < 0.01), as shown in Fig. 5A, indicating that DNA damage checkpoints play a key role in the induction of RAR by volatile stress signals. The DNA repair pathways located downstream of the DNA damage checkpoints also play an important role in the induction of RAR. As shown in Fig. 5B, a nucleotide excision repair (NER) pathway mutant *(xpf-1*), nonhomologous end joining (NHEJ) pathway mutants (*lig-4* and *ku80)*, mismatch repair (MMR) pathway mutant (*msh-6*), and homologous recombination (HR) pathway mutant (*brc-1*) were used for the experiments. The data illustrated that RAR induction was prevented in the NER pathway mutant (*xpf-1*), the NHEJ pathway mutants (*lig-4* and *ku80*), and the MMR pathway mutant (*msh-6*) (*P* > 0.05 for the *xpf-1, lig-4,* and *msh-6* genes). However, increased embryonic lethality was observed for the *cku-80* mutant (*P* < 0.05), whereas RAR induction still existed in the HR pathway mutant (*brc-1*). These results indicated that the DNA damage checkpoints NER, NHEJ, and MMR might all contribute to RAR induction by volatile stress signals.

### Regulation of the HSR and ER pathways in the production of volatile stress signals

**Figure 6:**
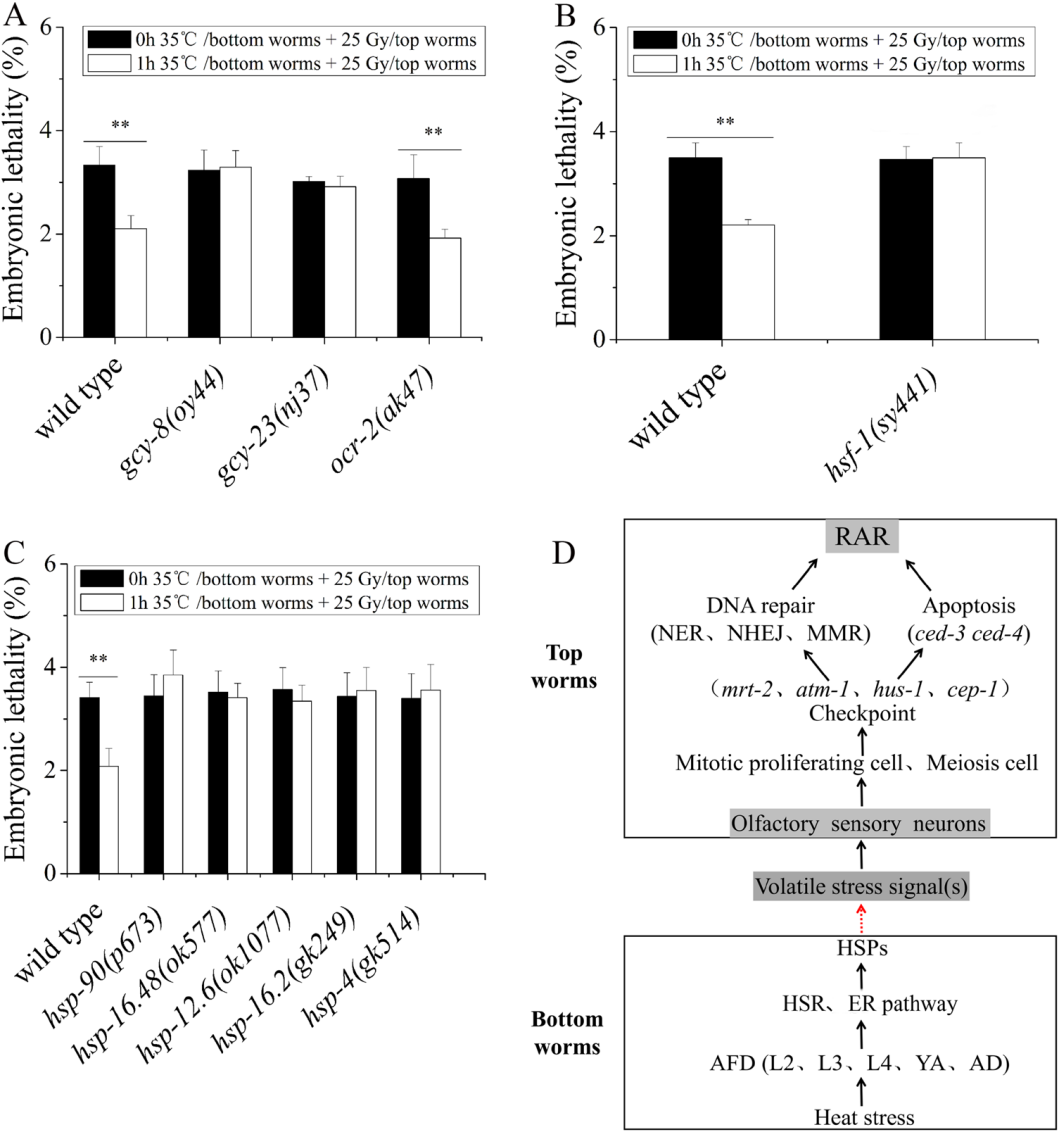
HSPs regulates the production of the heat-stress volatile signals in the bottom worms under heat stress. (**A**–**C**) Embryonic lethality of the top worms (N2) after coculture with heat-stressed bottom worms whose thermosensory neuron (*gcy-8, gcy-23*, *and ocr-2*)*, hsf-1,* and HSPs functions are absent; (**D**) Proposed model of production and action of heat-stress volatile signals. Bottom worms sense heat stress by AFD and activate HSF-1. HSPs activate downstream related genes to produce heat-stress volatile signals. Top worms sense volatile stress signals by olfactory sensory neurons to activate DDR pathway (checkpoint, DNA repair, apoptosis) and induce RAR in meiotic and mitotic proliferating zone of gonad. Results are means ± SD (n = 5, **P < *0.01*).

In *C. elegans*, ascarosides are prominent groups of chemical signals that mediate communication, including male attraction, hermaphrodite repulsion, olfactory plasticity, and aggregation, between worms of the same species^15–21^. Joo et al.^41^ showed that temperature affected the biosynthesis of ascarosides. To check whether ascarosides could contribute to the production of volatile stress signals induced by heat stress in the bottom worms, mutant worms (*acox-1*, *maoc-1*, *dhs-2*8, and *daf-22* genes), whose ascaroside biosynthesis function is absent, were used as the bottom worms in the coculture system. We found that the heat-stressed ascaroside biosynthesis mutants (*acox-1*, *maoc-1*, *dhs-2*8, and *daf-22* genes) still induced the RAR of embryonic lethality in the top worms (in all cases, *P* < 0.01) (Fig. S3), suggesting that the production of volatile stress signals is not related to the ascaroside biosynthesis pathway. The heat shock response (HSR), known as the cytosolic unfolded protein response, is activated in response to heat stress or other insults that disrupt protein folding in the cytoplasm. To cope with the abnormal rise in temperature that triggers protein misfolding and aggregation, *C. elegans* detects and responds to ambient temperature through AFDs to activate heat shock transcription factor-1 (HSF-1) in the cytoplasm. Subsequently, HSF-1 converts from an inactive monomer to an active trimeric form and activates the expression of heat shock proteins (HSPs), including molecular chaperones^42–45^. Therefore, we tested whether HSF-1 regulated the production of volatile stress signals induced by heat stress in the bottom worms. To do this, several loss-of-function mutations affecting AFD neurons, including HSF-1 and HSPs, were used as the bottom worms. As shown in Fig. 6A–C. AFD (*gcy-23 and gcy-8*), *hsf-1*, *hsp-90*, small HSP (*hsp-16.48, hsp-12.6, and hsp-16.2* genes), and HSP-4 (endoplasmic reticulum [ER] chaperone) mutations all inhibited RAR induction in the top worms (in all cases, *P* > 0.05), suggesting that the HSR and ER pathways have roles in controlling the production of volatile stress signals. However, only the *ocr-2* mutation did not affect the thermosensory function of the AFD neuron; instead, it affected the sensory function of four other neurons, ADF, AWA, ASH, and ADL, which still presented RAR induction in the top worms (in all cases, *P* < 0.05), further indicating that HSF-1 is essential in controlling the production of volatile stress signals.

## Conclusion

**Figure 7:**
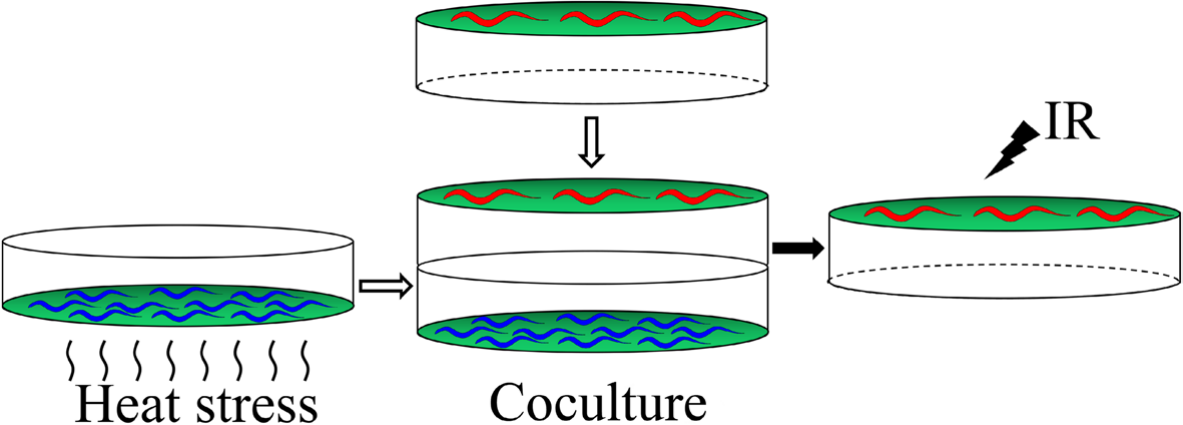
Schematic drawing of co-culture experimental system, in which red worms represent the top worms, blue worms represent the bottom worms, and the top worms communicate with the bottom via volatile signals.

To summarize, in this study, we demonstrated that nematodes are able to generate volatile stress signals when subjected to heat stress. To prove that *C. elegans* has long-distance communication through volatile stress signals under heat stress, we established a coculture experimental system (Fig. 7)*.* First, we used a Petri dish coated with only *E. coli* OP50 exposed to heat stress as the bottom dish in the coculture system and found that the production of volatile stress signals was inhibited (Fig. S2), which excluded the possibility that the volatile stress signals were derived from heat-stressed *E. coli* OP50. Second, when several loss-of-function mutations affecting HSF-1 and ascaroside biosynthesis were used as the bottom worms, the generation of volatile stress signals was blocked (Fig. 6A–C, Fig. S3), further indicating that the volatile stress signals were not derived from heat-stressed *E. coli* OP50 but were produced by heat-stressed nematodes. Third, RAR was prevented when worms whose olfactory sensory neuron function (*che-2, str-2, odr-3,* and *odr-1*) was absent were used as the top worms (Fig. 1B). Since the top worms received airborne signals only through olfactory sensory neurons, the data further suggested that heat-stressed nematodes could induce volatile stress signals. Finally, the RAR of the top worms could be induced only when the bottom worms were within the heat stress range (Fig. S1). Furthermore, we also found that RAR could be induced in the top worms when the bottom worms were subjected to high-density stress (16,000 worms per dish) (Fig. 2B). The above results showed that the worms under both heat stress and high-density stress can produce volatile stress signals. Heat stress and high-density stress are the two most common types of stress in nature, which means that *C. elegans* is likely to produce volatile stress signals for communicating stress cues over a long distance when under stress. Once an individual living organism is subjected to stress, volatile stress signals will spread throughout the entire population, inducing the entire population into a state of warning to decrease potential damage.

To survive in a changing environment, animals must be able to respond to the stresses encountered. The HSR is a highly conserved cellular pathway that is responsible for stress relief and the refolding of denatured proteins. It can be activated via several stressors such as an increase in temperature, oxidative stress, glucose depletion and/or the overexpression of misfolded proteins^46^. Our results confirmed that the HSR plays a role in controlling the production of volatile stress signals induced by heat shock (Fig. 6A–C). Our group has demonstrated that the production of radiation-induced volatile signals depends on ascarosides secreted by *C. elegans*^31^. In this study, we found that the production of volatile signals induced by heat stress is not related to ascarosides (Fig. S3), which means that volatile signals induced by heat stress and radiation stress are most likely two different types of substances. When we used HSF-1 mutants as heat-stressed bottom nematodes, we found that the generation of volatile stress signals was blocked (Fig. 6A–C), indicating that HSF-1 was involved in the regulation of volatile stress signals. When the HSF-1 mutant was used as the radiation-stressed bottom worms, RAR was still induced in the top worms (Fig. S4), which further suggests that the volatile signals induced by radiation were different from those induced by heat stress.

The ER is a site of protein synthesis that can become perturbed in response to misfolded proteins and/or ER calcium imbalance. Stress in this organelle induces the unfolded protein response (UPRER), which was previously shown to regulate longevity in a cell-nonautonomous manner in *C. elegans*^47,48^. HSP-4 is a chaperone in the ER that is part of the secretory pathway, but it is not involved in the heat shock response, which is found in the cytoplasm. Surprisingly, our results show that HSP-4 mutation can affect the production of volatile signals (Fig. 6C), which indicates that the ER pathway is probably involved in the regulation of signal production. Howard AC et al*.* showed that reducing translation through eIF4G/IFG-1 improves survival under ER stress and that this response depends on heat shock factor HSF-1 in *C elegans* ^47^. This result indicates that there is a certain correlation between HSF-1 and the ER stress response. Previous researchers reported that heat shock can increase the relative protein levels of the respective fusion protein (HSP-4::GFP) in *C. elegans*^49,50^. This result suggests that *C. elegans* responds to elevated temperature conditions by triggering not only the heat shock response but also the ER pathways. Although further investigation is required to determine how the HSR and ER pathways regulate the production of volatile signals, the results of the current study indicate that the HSR and ER pathways can influence the generation of volatile signals induced by heat shock.

Heat shock proteins (HSPs) are not volatile and therefore cannot be directly used as volatile signals induced by heat stress. Therefore, how HSPs regulate the production of volatile stress signals is unknown. Initially, HSPs were only considered to be molecular chaperones^51^, but increasing evidence now suggests that HSPs can not only be secreted in vitro^52^ but also directly activate the relevant downstream genes in cells^53^. Additionally, they can be secreted in the extracellular and cell-associated compartments to elicit a range of biological effects^54^. Here, to determine whether HSPs can also be secreted into the environment and sensed by nematodes to regulate the production of volatile stress signals, we used nematodes with chemosensory neuron impairments as the bottom worms and found that RAR did not disappear (Fig. S5), indicating that heat-stressed nematodes did not secrete HSPs in vitro. Therefore, we speculate that there are two possible ways for heat-stressed nematodes to regulate the production of volatile stress signals through HSPs. First, HSPs directly activate downstream signaling pathways to regulate the production of volatile stress signals. Second, HSPs bind to receptors on the surface of other cells in the body to initiate related signaling pathways that regulate the production of volatile stress signals. Therefore, exploring how HSPs regulate the production of volatile stress signals will be a focus of our future work.

*p53* is a key regulator of the DNA damage-induced checkpoint in mammals^55^ and is necessary to maintain gene stability and trigger apoptosis in abnormal cells that may become tumor cells^56–58^. Our results showed that *cep-1*, the *C. elegans* ortholog of the human tumor suppressor *p53*^59^, is necessary for the top worms to induce RAR (Fig. 5A). Ced-3 and ced-4 are essential core elements for the apoptosis pathway in *C. elegans*^60^. CED-3 is a member of the caspase family of proteases; CED-4 is homologous to mammalian Apaf-1 and is a positive regulator of CED-3^61^. Our results confirmed that the apoptotic pathway is involved in RAR induction by volatile stress signals in the top worms (Fig. 4A). At present, most human cancers evade *p53* tumor suppressor activity by selecting for mutations in *p53* itself^62,63^. This suggests that the volatile stress signals under heat stress are very likely to enhance the inhibition of cancer cells in mammals. Furthermore, our results indicate that the nucleotide excision repair (NER) pathway (*xpf-1*), nonhomologous end joining (NHEJ) pathway (*lig-4* and *ku80*), and mismatch repair (MMR) pathway (*msh-6*) are all involved in RAR induction by volatile signals in the top worms (Fig. 5B). These genes (*xpf-1, lig-4, ku-80*, and *msh-6*) are not only homologous to those in humans but also have highly conserved repair functions^39^. The volatile substances increase the radiation resistance of nematodes, indicating that they may also have a similar function in humans. According to reports, 60–80% of *C. elegans* genes have orthologs in the human genome^64^, which means that the substances secreted by nematodes themselves that induce radiation resistance are likely to have the same effect on humans. The chemical structure of the volatile signals induced by heat stress has not yet been identified. Further analysis of its structure might provide a new way to search for antiradiation drugs.

We canonically demonstrated that heat-stressed nematodes could communicate with unheated nematodes via volatile stress signals. While the regulatory pattern of signal production and action is preliminarily clarified, as shown in Fig. 6D, its chemical nature is unclear and should be the primary topic of further investigation.

## Materials and methods

### Worm strains and culture conditions

All *C. elegans* strains were cultured at 20 °C using standard conditions^65^, unless otherwise noted. The N2 Bristol strain was used as the wild-type strain. In addition, the following mutant strains were used in the genetic analyses: VC1785: *acox-1*(*ok2257*)I, VS18: *maoc-1*(*hj13*)II, VS8: *dhs-28*(*hj8*)X, and DR476: *daf-22*(*m130*)II, MT2547: *ced-4*(*n1162*)III and MT1522: *ced-3*(*n717*)IV, VC381: *atm-1*(*gk186*)I, SP506: *clk-2*(*mn159*)III, CB5348: *mrt-2*(*e2663*)III, WS2277: *hus-1*(*op241*)I, VC172: *cep-1*(*gk138*)I, TJ1: *cep-1*(*gk138*)I, RB873: *lig-4*(*ok716*)III, RB964: *cku-80*(*ok861*), RB1209: *brc-1*(*ok1261*)III, DW102: *brc-1*(*tm1145*)III, NL2511: *msh-6*(*pk2504*)I, CB1487: *xpf-1*(*e1487*)II, CB1033: *che-2*(*e1033*)X, VC2413: *str-2*(*ok3089*)V, CX2065: *odr-1*(*n1936*)X, CX2205: *odr-3*(*n2150*)V, CB1377: *daf-6*(*e1377*)X, PR808: *osm-1*(*p808*)X, MT3762: *osm-3*(*n1540*)IV, PR802: *osm-3*(*p802*)IV, PR811: *osm-6*(*p811*)V, IK800: *gcy-8*(*oy44*) IV, IK427: *gcy-23*(*nj37*) IV, CX4544: *ocr-2*(*ak47*) IV, PS3551: *hsf-1*(*sy441*) I, VC1099: *hsp-4*(*gk514*) II, VC475: *hsp-16.2*(*gk249*) V, RB1098: *hsp-12.6*(*ok1077*) IV, RB791: *hsp-16.48*(*ok577*) V, and PR673: *hsp-90*(*p673*) V. All of the nematode strains were from the *Caenorhabditis* Genetics Center (CGC).

### Heat stress treatment

Worms at the L3 stage were placed on 60 mm dishes containing NGM agar and bacteria at a population density of 400 worms per plate, and 3 independent plates were used. To avoid dehydration, plates were sealed with parafilm, packed into closed carton boxes, and then put into the Sanyo MIR-162 incubator that was running at 35 °C.

### Protocols for coculture of worms

The plates with worms were transferred to the incubator at 20 °C immediately following heat stress treatment. The heat-stressed worms were placed at the bottom of the coculture system, as shown in Fig. 7; other operating methods were described in Tang et al.^31^. After 6 h of coculture, the top worms were removed from the coculture system and then subjected to 25 Gy of irradiation. The irradiated worms were allowed to lay eggs for 36 h and then removed from the top Petri dishes. After 24 h, the number of unhatched eggs and hatched larvae (F1) on the top Petri dishes was counted under a dissection microscope to calculate embryonic lethality^40^. The final dataset consists of the averages of at least five independent experiments; approximately 900 eggs from six tested worms (with different degrees of reductions for certain mutant worms) were scored in each experiment.

### Gamma irradiation

The top worms were sealed with parafilm and exposed to gamma rays at 25 Gy and a dose rate of 3.37 Gy/min using a Biobeam Cs137 irradiator (cat no. GM 2000; Gamma-Service Medical, Leipzig, Germany). The temperature of the room was kept at 20 °C when the worms were irradiated.

### Statistical analysis

All experiments were repeated at least three times with identical or similar results. All results are presented as the means ± standard deviations. All comparisons for differences among two or more datasets were determined by performing Student’s *t test*, with *P* values < 0.05 considered to be significant.

## Supplementary Information

Supplementary Information.

## Data Availability

The datasets used and/or analysed during the current study available from the corresponding author on reasonable request.
