# Supplementary material for: Investigation into the communication between unheated and heat-stressed Caenorhabditis elegans via volatile stress signals
Source: Sci Rep. 2023 Feb 24;13:3225. doi: 10.1038/s41598-022-26554-8 (PMC9958180; doi:10.1038/s41598-022-26554-8)
Supplement: Supplementary file 1 — Supplementary Information. [file 41598_2022_26554_MOESM1_ESM.pdf]

Investigation into the communication between unheated and heat-stressed *Caenorhabditis elegans* via volatile stress signals

Liangwen Chen[a,b,c,1], Yun Wang[b,1], Xiuhong Zhou[a], Ting Wang[c], Huimin Zhan[b], Fei Wu[b], Haolan Li[b], Po Bian[c,*], Zhongwen Xie[a,*]

Supplementary data

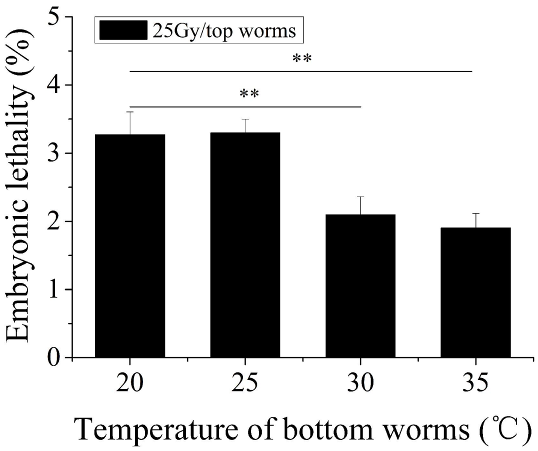

Supplementary figure 1. RAR of embryonic lethality in top worms was induced after coculture with bottom worms subjected to the indicated durations of heat exposure (30/35 ℃). Results are means ± SD (n = 5, ** $P < 0.01$).

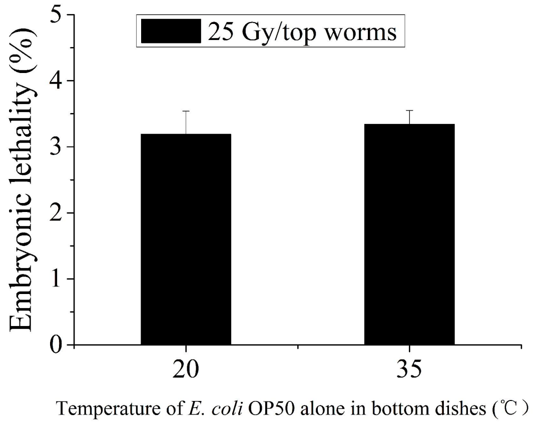

Supplementary figure 2. Heat-stressed *E. coli* OP50 in bottom dishes alone has no effects on the induction of RAR of embryonic lethality in top worms. Results are means ± SD (n = 5).

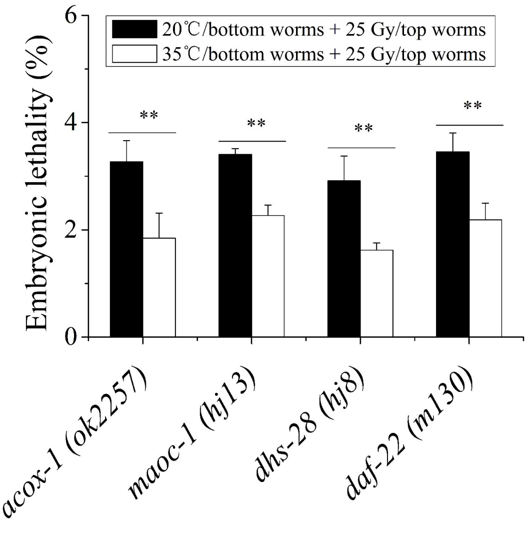

Supplementary figure 3. RAR of embryonic lethality in top worms was induced after coculture with heat-stressed bottom worms in which the ascaroside biosynthesis is impaired. Results are means ± SD (n = 5, ** $P < 0.01$).

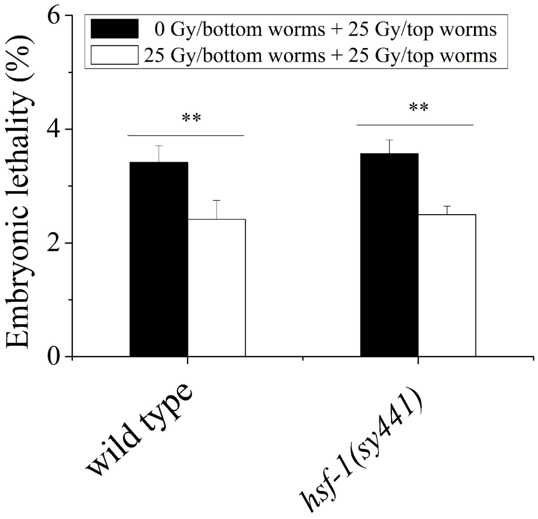

Supplementary figure 4. RAR of embryonic lethality in top worms was induced after coculture with radiation-stressed bottom worms structurally impaired the gene *hsf-1*. Results are means ± SD (n = 5, ** $P < 0.01$).

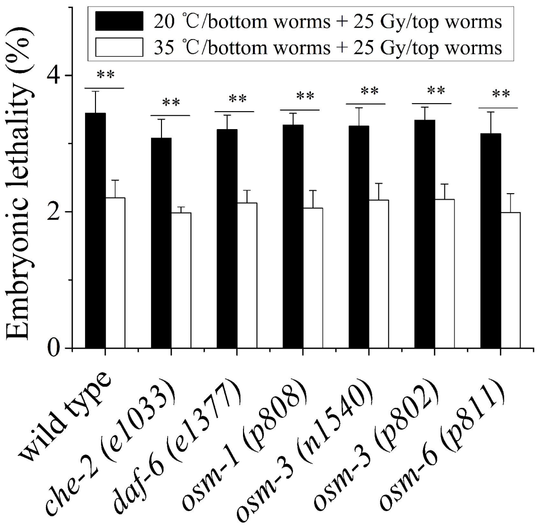

Supplementary figure 5. RAR of embryonic lethality in top worms was induced after coculture with heat-stressed bottom worms structurally impaired chemosensory neurons. Results are means ± SD (n = 5, ** $P < 0.01$).
